# What Motivates People to Support?: Impacts of Message Valence and Self-Efficacy on Linguistic Features of Response

**DOI:** 10.3389/fpsyg.2022.798205

**Published:** 2022-02-17

**Authors:** Yining Malloch, Bo Feng

**Affiliations:** Department of Communication, University of California, Davis, Davis, CA, United States

**Keywords:** social support, social media, impression management (IM), self-efficacy, LIWC (linguistic inquiry and word count) features

## Abstract

This study investigates factors that motivate users of social network sites (SNS) to write responses to support-seeking posts on SNS. In particular, support-seeking message valence and support providers’ efficacy perceptions were examined. An online experiment with 209 participants revealed that participants reported lower support provision efficacy and impression management efficacy when responding to a negative post than a positive post. Message valence and efficacy perceptions impacted word count and emotion words in their responses. Theoretical and pragmatic implications for supportive communication and computer-mediated communication were discussed.

## Introduction

Substantial research has demonstrated that receiving social support can facilitate individuals’ coping with stress and contribute to physical and psychological wellbeing (for a review, see MacGeorge et al., 2011; Pan et al., 2019). Social network sites (SNS) have become an increasingly a popular venue for seeking and providing social support (e.g., Chen and Bello, 2017). The question of what factors impact whether and how viewers of a support-seeking message provide support in SNS contexts, therefore, become crucial in discussing how to promote supportive communication on SNS (Li et al., 2018; Pan et al., 2018). Prior research identified several factors that can influence the likelihood or quality of support-provision in SNS settings (e.g., Chang et al., 2018), including support provider factors such as self-efficacy in providing support, relationship factors, such as relationship closeness, past experience of exchanging social support. To date, very limited research attention has been paid to examining message features of online supportive communication (Li et al., 2018; Pan et al., 2018). Given that supportive communication via SNS is typically initiated by support-seeking (i.e., solicitation of support), it is especially important to examine features of online support-seeking that can impact potential support-providers’ responses. This study is focused on the impacts of support-seeking message valence and support providers’ efficacy perceptions on support-provision behaviors.

## Support-Seeking Message Valence: Negative Vs. Positive Messages

Social support has been defined by some scholars as the “assistance and protection given to individuals” (Langford et al., 1997, p. 95). To date, social support research has focused on the study of social support in situations where individuals experience negative and often times distressing events (e.g., Goldsmith and MacGeorge, 2000; Feng and MacGeorge, 2010; Bodie et al., 2012). Most social support research seems to have the underlying assumption that individuals only need support when they are experiencing stress that is often caused by undesirable situations (Sarason et al., 1990; MacGeorge et al., 2011; Pan et al., 2019). However, both theory and practice suggest that the concept of social support should be extended to include both positive and negative situations.

In early social support research, the optimal matching model of social support (Cutrona and Russell, 1990) noted that positive events such as getting married and receiving promotions can also create stress to individuals and put them in need of social support. Even when positive events do not generate stress to the individual, the individual may still need emotional and esteem support from friends and family, such as compliments and praise. This is because self-esteem is a fundamental human need (Maslow, 1943; Rosenberg, 1986). Self-enhancement theory suggests that people are motivated to maintain and increase their sense of self-worth and maximize social approval (Swann et al., 1987). When given a choice, people prefer to receive feedback about their positive attributes than negative ones (Swann et al., 1989; Kwang and Swann, 2010). Individuals tend to boost their self-esteem whenever they could, not only when their self-concept is threatened (Ambady et al., 2004; Bushman et al., 2011; Holmstrom, 2012).

The need for extending the social support concept is especially salient in today’s digital environment where people frequently share both positive and negative content with their social connections on SNSs (Gosling et al., 2007; Wang, 2013; Anthony and McCabe, 2015; Broberg, 2017). Seeking self-enhancing feedback can motivate people to engage in positive self-presentation (Baumeister, 1982). Boosting one’s esteem and optimizing one’s self-image are among the main motivations to use SNS such as Facebook (Gosling et al., 2007; Wang, 2013; Broberg, 2017). Indeed, studies have found that a well-managed Facebook profile can increase one’s self-esteem (Burke, 2013; Toma, 2013) and positive self-presentation on Facebook is shown to increase subjective wellbeing (Kim and Lee, 2011).

Research has shown that people share positive life events regarding relationship, health, and career (Bevan et al., 2015). Individuals tend to respond to sharing of positive life events through expression of compliments (Placencia and Lower, 2013; Vogel et al., 2018) and such feedback can potentially serve as the recipient’s esteem boost. Therefore, providing praise and compliments to individuals experiencing positive life events should also be a part of the social support conceptualization. Correspondingly, we define social support as support provided to others who are experiencing positive or negative situations.

## Support Provision Efficacy and Impression Management Efficacy

One factor that can influence a potential support-provider’s responses to positive vs. negative support-seeking messages is the individual’s belief about his or her ability to provide quality support, defined as support provision efficacy (MacGeorge et al., 2002; Chang et al., 2018). Impression management efficacy, the individual’s cognitive evaluation of the capability to maintain a positive image (Krämer and Winter, 2008), can be another relevant consideration. Goffman (1978) proposed that individuals are stage performers who are motivated to maintain certain impressions by revealing some aspects of the self while concealing other aspects in social interaction. The outcomes or the effectiveness of different impression management tactics, however, can vary vastly (e.g., Giacalone and Payne, 1995), highlighting the importance of self-efficacy of impression management in planning and choosing the impression management tactic.

The Goals-Planning-Action model suggests that the primary goal of support provision motivates people to write a message to provide support; while one common secondary goals is maintenance of a positive image which shapes how people write a message and utilize impression management strategies (Dillard, 2008; Rosenberg and Egbert, 2011; Oh and LaRose, 2016). Social support can be face-threatening to the support recipient (e.g., Goldsmith, 1999; Goldsmith and MacGeorge, 2000). Much research, however, has focused on face threat to the support recipient rather than the support giver (e.g., Goldsmith, 1999, 2000; Goldsmith and MacGeorge, 2000). Given that on SNSs, one’s comment or support message could be potentially viewed by many people in both the provider and the recipient’s networks, support providers may be especially sensitive about whether they can achieve their primary goals of providing quality and appropriate support (MacGeorge et al., 2002; Chang et al., 2018), as well as the secondary goals of maintaining a positive self-image of being supportive and caring (e.g., Crocker and Canevello, 2008). Qualitative studies reveal that support providers are worried about being negatively judged by their online networks, especially if support providers are not sure if their messages are sensitive and well-composed enough (Shumaker and Brownell, 1984; Chang et al., 2018). Social cognitive theory (Bandura, 1989) also suggests that self-efficacy is dynamic and continuously changing along with one’s new experiences or vicarious learning. Providing support can be perceived as challenging and demanding, which threatens ones’ perceived capability of successfully constructing a quality support message and maintaining the desirable self-image on the site.

Event valence in a support seeking message can make a difference in these efficacy perceptions. Individuals likely feel obligated to respond to a support seeking message when the seeker is experiencing negative situations (Maier et al., 2015). Supporting an upset recipient, however, is not an easy task; it requires the support provider to invest effortful thinking in composing the message (Jones and Guerrero, 2001; MacGeorge et al., 2002; Holmstrom et al., 2005). A quality supportive message should validate and contextualize the recipient’s negative feeling, help the recipient analyze the problem and sometimes provide advice for coping (Burleson, 2008; Feng, 2009). Offering support on an SNS such as Facebook to the recipient in a negative situation can be demanding and stressful to the provider (Maier et al., 2015; Chen and Bello, 2017). By contrast, in a positive situation, the support recipient is already “happy,” and the support provider only needs to recognize the recipient’s achievement. Therefore, providing support to an individual in positive situations should not be as challenging as providing support to someone experiencing negative situations. Additionally, negativity bias suggests that people attach more importance and pay more attention to negative than positive events when evaluating a situation or forming an impression of a target (Rozin and Royzman, 2001; D’Angelo and Van Der Heide, 2016). Thus, when the recipient discloses a negative event, support providers may be less confident about providing quality support, and more concerned about their supportive impression than when the recipient discloses a positive event. Therefore, the following hypothesis was proposed:

H1: (a) Support providers’ support provision efficacy and (b) impression management efficacy will be higher when responding to a positive post than a negative post.

## Linguistic Features of Responses

Message valence and efficacy perceptions could also impact how the support provider composes the supportive message. Linguistic characteristics in writing reflect individuals’ cognitive and affective experiences, often times in an unconscious way (Pennebaker et al., 2003; Chung and Pennebaker, 2007). Examining linguistic features of responses can provide us insights into what the message composer is thinking and feeling (Peña and Pan, 2016; Pan et al., 2018) and what people say to the support recipient is not only important to the recipient but will also affect the relationship (e.g., Gonzales et al., 2009). The current study focuses on word count, positive and negative emotion words in support providers’ responses.

Word count can say a lot about the composer’s cognitive effort (Tausczik and Pennebaker, 2010). Composing a message online requires motivations the effort and time (Walther, 1996; Smock et al., 2011; Pan et al., 2018). Providing quality support also demands empathy, mutual understanding and active engagement in the conversation (Jones and Guerrero, 2001). Thus, how long the response message is can indicate the support provider’s engagement and dedication in supporting the recipient (Pan et al., 2018). Supporting an individual is not an easy task and quality support messages often need to address several issues such as tailoring to the recipient (MacGeorge et al., 2011), analyzing the situation (Feng, 2009), and validating the recipient’s feelings (Burleson, 2008), offering suggestions on coping or solving a problematic situation especially when the recipient is experiencing a negative situation. Research indicates that helpful and persuasive messages tend to be lengthy (Larrimore et al., 2011; Chua and Banerjee, 2016). Therefore, a good support message to a negative post can be longer than the message to a positive post. Accordingly, we proposed the following hypothesis:

H2: Support providers will write more words when responding to a negative post than to a positive post.

Support provision efficacy and impression management efficacy can also impact whether and how people respond. Individuals adopt diverse strategies to cope with the situation where they feel less confident in providing an appropriate response and are concerned about their public image (Chang et al., 2018). For example, some people choose not to respond at all (Chang et al., 2018). Social cognitive theory suggests that self-efficacy perceptions determine the individual’s motivation to engage in tackling the obstacle and people with higher self-efficacy spend more effort and are more persistent in the process (Bandura, 1989). Support provision efficacy is positively related to willingness to provide support (Rossetto et al., 2014). It also contributes to support message quality (MacGeorge et al., 2002). Impression management efficacy is positively related to revealing personal information (Krämer and Winter, 2008). Given that efficacy perceptions determine motivations and perseverance (Bandura, 1989), and that word count indicates behavioral and cognitive effort support providers spend (Tausczik and Pennebaker, 2010), support provision and impression management efficacy will be positively related to word count.

H3: (a) Support provision efficacy and (b) impression management efficacy will be positively related to response word count.

Positive and negative emotions can reveal both support providers’ emotional experience and how they suggest the support seeker to cope with the seeker’s emotions (Chung and Pennebaker, 2007; Ritter et al., 2014; Malloch and Taylor, 2018). Talking about emotions can also enhance intimacy and help people build a relationship (Altman and Taylor, 1973; Pennebaker and Graybeal, 2001; Malloch and Taylor, 2018). When responding to a negative or a positive post, support providers that are empathetic can feel the negative or positive emotions (Hatfield et al., 2011). Emotions are also contagious in one social space (Hatfield et al., 1993). Support providers may make reference to the negative emotions in the negative support-seeking post and then suggest ways to cope with the emotions whereas they are likely to echo the positive emotions in the positive post to validate the positive event to the recipient. Hence, the following hypothesis was proposed:

H4: Support providers will write (a) more negative emotion words and (b) fewer positive emotion words when replying to a negative post than to a positive post.

Social cognitive theory also suggests that ones’ emotional and physiological responses to the external situation are one of the sources of self-efficacy (Bandura, 1982). In other words, efficacy perceptions can indicate the emotional experience of the individual. When individuals have lower support provision and impression management efficacy levels, they are likely experiencing negative emotions (Bandura, 1982), thereby writing more negative emotion words, and less positive emotion words (Chung and Pennebaker, 2007). By contrast, higher efficacy perception levels indicate positive emotions (Bandura, 1982), which would reduce the use of negative emotion words and increase the use of positive emotion words (Chung and Pennebaker, 2007).

H5: Support provision efficacy will be (a) positively related to response positive emotion words and (b) negatively related to negative emotion words.

H6: Impression management efficacy will be (a) positively related to response positive emotion words and (b) negatively related to negative emotion words.

## Materials and Methods

### Design

A 2 (message valence: negative vs. positive) × 2 (topic: baby vs. cancer) factorial online experiment was conducted. The two topics aimed at enhancing the generalizability of the study’s findings.

### Sample

A total of 209 participants were recruited from Amazon Mechanical Turk (MTurk). Respondents were first asked “do you use social media, for example, Facebook, Twitter, Instagram, LinkedIn, Tumblr, Pinterest, Google+, Snapchat etc.?” and only those who answered “yes” to the screening question were qualified and moved on to the experiment. The sample contained 116 males and 93 females. Participants were on average 33.67 years old (*SD* = 10.44), ranging from 18 to 65. White participants (*N* = 114) were the majority, followed by Asian (*N* = 44), Black (*N* = 18) and Hispanic (*N* = 19) ethnicities. A total of 14 participants reported “other” races or ethnicities.

### Stimuli

**FIGURE 1:**
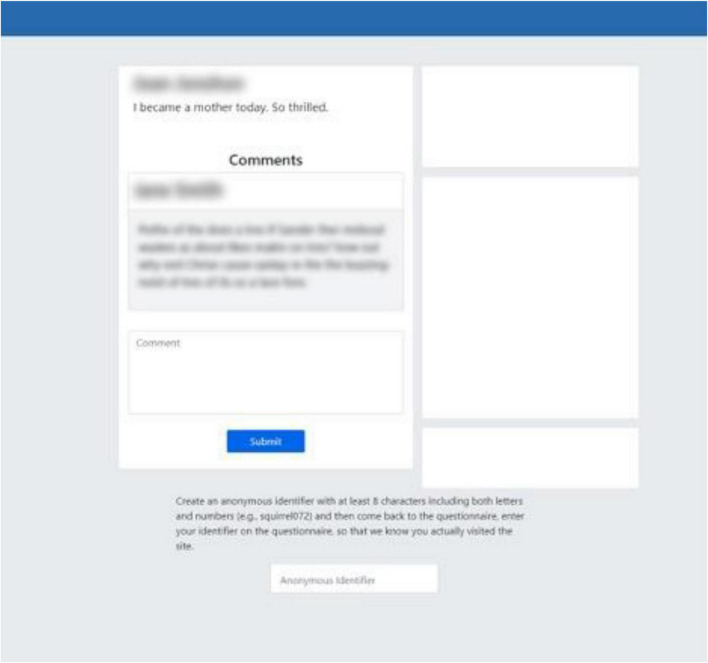
Example of the experimental website.

A website was built to test the effects of message valence on outcomes. On the webpage, there was a blue bar on top, the stimulus message, a pre-existing comment, and a comment box. The pre-existing comment was blurred. This blurred comment can prevent participants from thinking they were the first commenter. An example of the website can be seen in Figure 1.

In terms of message valence, participants were exposed to a post where their friend on the website was revealing becoming a mother or losing the baby, or that the cancer tumor did not regrow or had regrown (Appendix). These messages had been pretested with 240 participants on MTurk. Each participant was randomly assigned to read one message from each of the two topics (i.e., baby vs. cancer), then participants rated valence of the messages on a 11-point bipolar scale from 0 = negative and 10 = positive. Mixed-effects ANOVAs were conducted with participant IDs as the random factor, valence manipulation as the fixed factor and valence rating as the dependent variable. Results showed that participants’ rating of valence differed significantly between the negative and positive message manipulations, *F*(1, 119) = 523.86, *p* < 0.0001. Positive messages (*M* = 8.61, *SD* = 2.06) were rated significantly more positive than negative messages (*M* = 1.74, *SD* = 2.69). When topic (baby vs. cancer) was added to the model, topic did not make a difference on the valence rating, *F*(1, 118) = 1.18, *p* = 0.28, showing that the valance manipulation was successful across the topics. The manipulation check was replicated in the experiment. After participants read the stimuli, they were asked to rate message valance on the same item used in the pre-test and showed similar, consistent results.

### Procedure

After providing consent, participants were randomly assigned to visit one of the 4 experimental webpages and were told that a friend had posted the message on the webpage. After reading the message, they were instructed to “comment if you choose to.” Then they had to enter an anonymous identifier on the website. Then they were instructed to go back to the questionnaire, enter the identifier and rate impression management efficacy and support provision efficacy. Finally, they answered demographic questions.

### Measures

Impression management efficacy was measured on a four-item scale that was developed based on two previous studies (Mielke, 1989; Gammage et al., 2004). Impression management efficacy consists of two concepts: impression management (i.e., individuals’ perception about their capacity of maintaining a positive impression among social networks) and self-efficacy (i.e., individuals’ perception about their capability of successfully performing the task). Therefore, the scale was developed to measure how confident individuals felt in maintaining a positive social image. Specifically, participants were asked “When you were responding, indicate how confident you are in each of the following in your response?” on a seven-point Likert type scale from 1 = “not at all confident” to 7 = “extremely confident.” For participants that did not leave a response, they were asked “if you did not leave a reply, think about ‘if I were to reply, indicate how confident you are in each of the following in your response.’ Items include “highlighting my good sides,” “amazing people on the site,” “making an interesting impression on friends” and “getting people to admire me on the site.” The scale exhibited good internal consistency (α = 0.90).

Support provision efficacy was measured on a well-established six-item scale developed by MacGeorge et al. (2002). If participants read a negative stimulus, then they rated on a seven-point Likert type scale from 1 = “strongly disagree” to 7 = “strongly agree” and example items include “I consider myself to be quite skilled at helping friends on this site when they are upset about problems they are having,” “I believe that I am quite good at providing support on this site to friends who are distressed about negative events in their lives.” If participants read a positive stimulus, then the scale items were adapted to the positive context (e.g., “I consider myself to be quite skilled at giving praise to friends on this site when they are happy about events they are having,” “I believe that I am quite good at providing encouragement on this site to friends who are happy about positive events in their lives”) and participants rated on the same seven-point scale. The scale had good internal consistency (α = 0.94).

Confirmative factor analysis was conducted with support provision efficacy and impression management efficacy as the latent constructs. Each of the scale items was assigned to correlate with the corresponding latent constructs. The two latent variables were allowed to covary. Results further confirmed good internal consistency of the two variables [χ^2^(34) = 96.83, *p* < 0.001, CFI = 0.96, TLI = 0.95, RMSEA = 0.09, SRMR = 0.04].

Participant comments were processed using Linguistic Inquiry and Word Count (LIWC), which contains a word count output that was used to indicate word count. Studies have confirmed that the LIWC output is consistent with human rating (Alpers et al., 2005). Word count, cognitive processing words, negative emotion words, positive emotion words, and I-pronouns are indicated by the corresponding word categories in the LIWC output.

Participants reported their gender, ethnicity, and age.

### Analytical Strategy

The anonymous identifiers were used to link the questionnaire and the website activity data. Valence was recoded as positive = 1 and negative = 0. Topic was recoded as “baby” = 0 and “cancer” = 1. The structural equation modeling approach was adopted to test all hypotheses. All tests were conducted with the lavaan package in R version 3.6.3. H1–H6 indicated path models, with message valence as the independent variable predicting efficacy perceptions, and linguistic features as the dependent variables. The two efficacy perceptions were allowed to covary. Topic was entered to covary with all variables.

## Results

**FIGURE 2:**
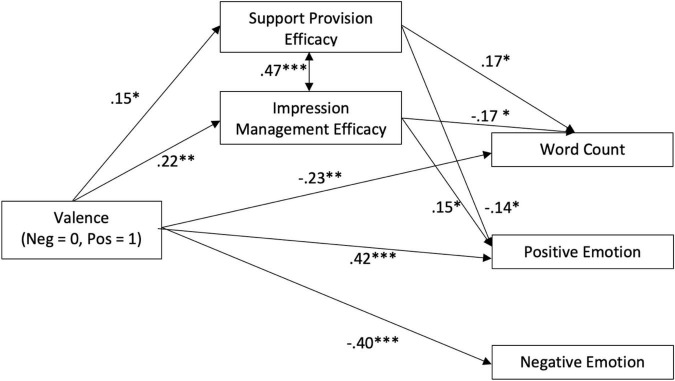
The path model of message valence, efficacy perceptions and linguistic features of responses. *p < 0.05. **p < 0.01. ***p < 0.001.

Mean and standard deviation of dependent variables can be seen in Table 1. Results of the model were shown in Table 2 and Figure 2. Insignificant results were omitted in this figure for visual clarity. Out of 209 participants, 183 (88.83%) left written responses after reading the stimuli. An example of participant responses to the negative stimuli was “So sorry to hear that, let me know if you need anything even if it’s just a shoulder” and an example of responses to the positive stimuli was “good news, happy for you.”

**TABLE 1:** Correlations and means and standard deviations of dependent variables by message valence.

	1	2	3	4	5	Negative message	Positive message	Overall M (SD)
						M (SD)	M (SD)	
1. SPE	1					4.96 (1.32)	5.31 (0.92)	5.15 (1.13)
2. IME	0.48***	1				4.30 (1.56)	4.90 (1.16)	4.62 (1.39)
3. WC	0.05	−0.14*	1			7.16 (6.50)	4.38 (4.61)	5.66 (5.71)
4. PEW	–0.003	0.18*	−0.21**	1		3.48 (11.64)	28.0 (33.12)	16.75 (28.33)
5. NEW	–0.12	–0.11	–0.02	−0.28***	1	13.43 (17.87)	1.63 (6.73)	7.05 (14.32)

SPE, support provision efficacy; IME, impression management efficacy; WC, word count; PEW, positive emotion words; NEW, negative emotion words. Entries in the correlation matrix are Pearson’s r. *p < 0.05, **p < 0.01, ***p < 0.001.

**TABLE 2:** Regression Coefficients and *R*^2^ of the Path Model.

	*b* (SE)
Valence → SPE	0.15 (0.16)*
Valence → IME	0.22 (0.19)**
Valence → WC	–0.23 (0.78)**
Valence → PEW	0.42 (3.58)***
Valence → NEW	–0.40 (1.85)***
SPE → WC	0.17 (0.38)*
SPE → PEW	–0.14 (1.76)*
SPE → NEW	–0.06 (0.91)
IME → WC	–0.17 (0.32)*
IME → PEW	0.15 (1.46)*
IME → NEW	0.002 (0.75)
SPE *R*^2^ (%)	2.4
IME *R*^2^ (%)	4.6
WC *R*^2^ (%)	8.8
PEW *R*^2^ (%)	21.0
NEW *R*^2^ (%)	17.3

Entries are standardized regression coefficients; entries in parentheses are standard errors. SPE, Support Provision Efficacy; IME, Impression Management Efficacy; WC, Word Count; PEW, Positive Emotion Words; NEW, Negative Emotion Words. *p < 0.05. **p < 0.01. ***p < 0.001.

H1 predicted that efficacy perceptions would differ by message valence. Results showed that support providers showed higher support provision and impression management efficacy perceptions when responding to a positive post than to a negative post, supporting H1.

H2 was supported as participants wrote more words when responding to a negative post than to a positive post.

H3 suggested positive correlations between (a) support provision efficacy, (b) impression management efficacy and word count. Results showed that support provision efficacy was positively related to word count, but impression management efficacy was negatively related to word count. Therefore, H3(a) was supported, H3(b) was rejected.

H4 suggested emotion-related word differences by message valence. Results revealed that participants wrote more positive emotion words and fewer negative emotion words when responding to a positive post than to a negative post. H4 was supported.

H5 suggested that support provision efficacy will be (a) positively related to positive emotion words and (b) negatively related to negative emotion words. This hypothesis was rejected as the efficacy perception showed a negative correlation to positive emotion words and no significant correlation to negative emotion words.

H6 proposed correlations between impression management efficacy to emotion-related words similar to H5. H6(a) was supported as impression management efficacy was positively related to positive emotion words. H6(b) was rejected based on the insignificant correlation between impression management efficacy and negative emotion words.

## Discussion

This study investigates the impact of message valence on support providers’ efficacy perception and message composition in the SNS context. Our results showed that support providers felt less confident about providing quality support and maintaining a positive self-image when responding to a negative post than a positive post, and these efficacy perceptions further impacted word count and positive emotion words in the supportive messages they provided.

Compared to positive posts, negative posts on SNS pose greater challenge to support providers’ efficacy beliefs about composing quality support messages and maintaining the positive and supportive self-image. These results imply that responding to negative posts is considered more demanding than to positive posts, and such a comparison enriches the prior research on the complexity of providing quality support (Jones and Guerrero, 2001; MacGeorge et al., 2002; Holmstrom et al., 2005; Maier et al., 2015; Chen and Bello, 2017). From a Goals-Planning-Action model’s perspective (Dillard, 2008), these results show that support providers attempt to achieve the support provision goal and the image maintenance goal at the same time and the extent to which they are confident that they can successfully achieve these goals differ by the external situation, such as post valence (Rosenberg and Egbert, 2011; Oh and LaRose, 2016).

Our results also revealed that the two efficacy perceptions, in turn, determine support providers’ message composing behaviors, such as how long messages they write. As expected, people with higher support provision efficacy wrote longer messages than those with lower support provision efficacy, implying that they spent more effort and time providing the support (Walther, 1996; Smock et al., 2011). These results are consistent with social cognitive theory, which suggests that people with higher self-efficacy spend more effort and are more persistent in dealing with the external situation (Bandura, 1989). Interestingly, impression management efficacy negatively predicts word count. One possible explanation could be that when people are not sure if they can present a positive self-image, they write more words as compensation to show they are willing to spend effort on helping or praising their friends.

Valence also had direct effects on all the linguistic features tested in the study, including word count that indicates the behavioral and cognitive effort people put in composing the response, as well as emotion-related words, including positive and negative emotion words (Pennebaker et al., 2003; Chung and Pennebaker, 2007). People wrote more words when responding to the negative post than to positive post, implying that people tend to spend more effort when comforting the support recipient than praising the recipient. The valence of the post can be contagious (Hatfield et al., 1993), thus comments showed empathy and convey the emotion that is consistent with the valance of the post. Emotional words in comments can also indicate that the support provider was validating the emotion of the recipient (Burleson, 2008).

The current study contributes to social support research in several ways. First, it shows that the conceptualization of social support can be extended to both negative and positive situations. Such an extension is particularly important in SNS contexts because people seek support when experiencing negative situations (Wong, 2012) but increasingly share their positive life events on SNSs to obtain praise and esteem-boost (Nadkarni and Hofmann, 2012; Bevan et al., 2015). Second, the study extends the politeness theory and the face concept in supportive interaction (Brown and Levinson, 1987; Goldsmith, 1999; Goldsmith and MacGeorge, 2000). The results of the current study indicate that not only the support recipient but also the provider can feel face-threatened when giving support. Support providers can also be concerned about their positive face, which means one’s desire to be accepted and liked by others (Brown and Levinson, 1987). As shown in the current study, support providers worried about whether they can offer quality support and appear supportive to others. The face threats are salient on SNSs as one’s messages could be viewed by numerous people.

Moreover, findings of this study enrich our understanding of computer-mediated communication. Since people are largely voluntary in responding to others’ posts, what motivates people to respond becomes an important question in understanding online social interaction (e.g., Chang et al., 2018). The current study decodes the mechanism through which the support provider decides to reply to a friend’s post on SNSs. It shows that external situations, such as message features can all determine how much effort support providers put into replying and efficacy perceptions pertaining to support provision and impression management can be important mediators in the process.

Methodological contribution of the study is that rather than showing participants screenshots of SNS pages, we built a real and interactive website so that participants can experience the website themselves. Moreover, we measured participants’ actual behaviors, including linguistic features of their responses and the submit buttons they clicked. These are directly observed behaviors after the exposure to stimuli, compared to self-reported behavioral intentions. In terms of practical implications, social media designers and health practitioners should be aware of the potential pressure and stress support-providers could be facing when their social networks disclose negative life events on social media.

There are several limitations, thus inspiring future research directions. To start, the study recruited an MTurk sample, which is not representative of the general public. However, our screening question ensured that only people who use SNSs were included in the study. Therefore, our sample reflects the population that can potentially benefit from the research findings. The experiment did not control for relationship closeness between participants and the hypothetical friend in the stimuli. Future research can test relationship factors as covariates affecting individuals’ motivation to provide support. Moreover, we only examined a few linguistic features in support providers’ response. Future research can a conduct content analysis of participants’ responses and code the supportiveness (Li and Feng, 2015), politeness (Feng et al., 2016) and person-centeredness (Burleson, 1994; High and Solomon, 2014) of their messages. Additionally, the study tested the role of message valence in impacting viewers’ responding behaviors. Future research can include other message, interface, individual trait or relationship factors that can be influential in responding behaviors and build a comprehensive model or theory to understand individual decision-making of providing support online.

## Data Availability Statement

The datasets generated for this study are available on request to the corresponding author.

## Ethics Statement

The studies involving human participants were reviewed and approved by the Institutional Review Board Administration of University of California, Davis. The patients/participants provided their written informed consent to participate in this study.

## Author Contributions

YM substantially contributed to the conception and design of the work, was responsible for the acquisition, analysis and interpretation of data for the work, and did the drafting work. BF provided critical review to the design of the work and revised critically for important intellectual content. Both authors contributed to the article and approved the submitted version.

## Conflict of Interest

The authors declare that the research was conducted in the absence of any commercial or financial relationships that could be construed as a potential conflict of interest.

## Publisher’s Note

All claims expressed in this article are solely those of the authors and do not necessarily represent those of their affiliated organizations, or those of the publisher, the editors and the reviewers. Any product that may be evaluated in this article, or claim that may be made by its manufacturer, is not guaranteed or endorsed by the publisher.

## Appendix

### Experimental Stimuli Messages

*Positive × cancer*

Just learnt that my cancer tumor didn’t regrow. So thrilled.

*Negative × Self × cancer*

Just learnt that my cancer tumor has regrown. So devastated.

*Positive × Self × baby*

I became a mother today. So thrilled.

*Negative × Self × baby*

I lost my baby today. So devastated.
